# Reflection as Evidence: Using Self-Perception to Assess the Impact of Faculty Development Programs on Teaching Competencies and Professionalism

**DOI:** 10.7759/cureus.86392

**Published:** 2025-06-19

**Authors:** Naeem Naqi, Sumreena Mansoor, Tatheer Zahra, Saba Iqbal, Zaima Ali

**Affiliations:** 1 Medical Education, Lahore Medical and Dental College/Lahore University of Biological and Applied Sciences, Lahore, PAK; 2 Biochemistry, Shifa Tameer-E-Millat University Shifa College of Medicine, Islamabad, PAK; 3 Anatomy, Shifa Tameer-E-Millat University Shifa College of Medicine, Islamabad, PAK; 4 Medical Education, Shifa Tameer-E-Millat University, Islamabad, PAK; 5 Health Professions Education, CMH (Combined Military Hospital) Lahore Medical College and Institute of Dentistry, Lahore, PAK; 6 Physiology, Lahore Medical and Dental College/Lahore University of Biological and Applied Sciences, Lahore, PAK

**Keywords:** career development, competency, faculty, faculty development program, medical education

## Abstract

Background

Faculty development programs (FDPs) play a pivotal role in enhancing the teaching competence and professionalism of the educators in higher education. These programs promote reflective practice and continuous learning and empower faculty members to adapt to evolving pedagogical demands and contribute meaningfully to academic excellence. This study investigates the faculty’s perceptions regarding the effectiveness of FDPs across five domains: institutional benefit, career development, competency motives, professional development, and faculty resilience and innovation.

Methodology

This single-center, cross-sectional study was held at the Combined Military Hospital Medical College Lahore and the Institute of Dentistry (CMH Lahore & IOD) over a nine-month period, after approval from the Ethical Review Board. Data were collected using a validated, reliable questionnaire (Cronbach’s alpha = 0.822), and quantitative analysis was performed by SPSS Version 22.0 (IBM Corp, Armonk, NY). The sample size was calculated using the online open-source and free software OpenEpi using the equation for a cross-sectional descriptive study. Descriptive statistics were used to summarize demographic data, and non-parametric tests were applied to assess differences across subgroups. The chi-square test was applied to check the association between the perception of teaching performance and variables such as professional experience, gender, and level of education. Binary logistic regression was used to identify predictors of perceived improvement in teaching performance. A total of 153 faculty members participated in the study.

Results

Most faculty members perceived that FDPs had a positive influence on career development, competency enhancement, and overall professional growth. Furthermore, there was a strong consensus among all participants that FDPs are essential for the benefit of the institution as well as faculty resilience and innovation. Male faculty members reported higher median scores in the domains of career development and professional development, indicating notable gender-based differences in the perception of FDP effectiveness. Faculty members with more than seven years of professional experience reported significantly higher scores in the domains of career development, professional development, and competency motives (p < 0.05). Binary logistic regression revealed that professional experience was a significant predictor of the perception that FDPs improve teaching performance (OR = 0.004, 95% CI: 0.001-0.016, p < 0.001).

Conclusion

FDPs foster innovation and play a vital role in enhancing faculty competence and improving teaching performance, particularly among experienced educators. Faculty members have a positive perception of the effectiveness of FDPs across five domains: institutional benefit, career development, competency motives, professional development, and faculty resilience and innovation. The findings underscore the influence of faculty experience on FDP outcomes and support the implementation of targeted, evidence-based faculty development initiatives. A sustained, context-sensitive, and outcome-driven approach is essential for optimizing the long-term impact of FDPs in medical education.

## Introduction

Faculty development programs (FDPs) play a crucial role in equipping educators with the skills and knowledge necessary to meet the ever-evolving demands of medical education [1]. These initiatives help educators refine their teaching methodologies, adopt innovative practices, and improve student engagement. Studies suggest that FDPs enhance teaching effectiveness by fostering professional growth and self-efficacy among faculty members, resulting in improved learning outcomes for students and increased institutional success [2,3]. Despite their importance, many institutions face challenges in implementing FDPs effectively. Limited financial resources, competing priorities, and a lack of awareness about the long-term benefits often prevent institutions from prioritizing these programs [4]. Furthermore, while faculty development promotes innovation and adaptability, some argue that it may shift focus and resources away from more critical areas, such as curriculum development and student support services [4]. Nevertheless, evidence highlights the potential of FDPs in a culture of continuous improvement, making them a vital component for faculty development and institutional success [5].

There is a need to better understand the effectiveness of FDPs in bridging the gap between program design, implementation, and institutional outcomes. Research has shown that access to professional development programs and opportunities enables faculty members to remain updated with technological advancements, academic trends, and innovative pedagogical techniques [6]. While the theoretical benefits of FDPs are widely acknowledged, empirical evidence regarding their perceived effectiveness from the faculty's perspective remains limited. Moreover, concerns that these programs may divert attention from essential academic functions highlight the importance of evaluating their practical impact [4]. In this context, understanding faculty perceptions offers a valuable lens through which to assess the alignment between FDP objectives and actual faculty needs. Despite the growing emphasis on continuous improvement in higher education, few studies have investigated how FDPs are experienced and valued by educators within diverse institutional cultures [5]. This gap is particularly pronounced in the Pakistani context, where faculty development is gaining momentum but lacks systematic evaluation across defined domains. In the context of resource-limited settings in Pakistan, evaluating the impact of FDPs becomes particularly important to ensure optimal allocation of institutional resources and faculty motivation. The current study was designed to assess the perceptions of faculty regarding the effectiveness of FDPs across five domains: institutional benefit, career development, competency motives, professional development, and faculty resilience and innovation. In this study, "effectiveness" refers to the extent to which faculty perceive FDPs to positively influence their professional roles across these five domains.

Each domain represents a critical aspect of faculty growth: institutional benefit focuses on contributions to the organization; career development involves personal advancement; competency motives reflect improvements in teaching ability; professional development emphasizes skill enhancement; and resilience and innovation address adaptability and creativity in academic roles. Self-perception was selected as the primary measure in the current study, as it provides a practical and meaningful insight into how faculty members internalize and evaluate the benefits of FDPs, especially in settings where standardized tools for assessing teaching outcomes or student performance may not be feasible. Faculty perceptions also serve as an early indicator of program relevance and areas needing improvement. Moreover, the study aimed to examine differences based on gender, education, and experience, and determine whether professional experience predicts perceived improvement in teaching performance.

## Materials and methods

Study design

This descriptive cross-sectional study was conducted at the Combined Military Hospital Medical College and the Institute of Dentistry over a nine-month period.

Study participants, duration, and sample size calculation

The study targeted faculty members from various academic departments within the institution. The sample size of the current study was 154 faculty members, calculated using the online open-source and free software OpenEpi using the equation for a cross-sectional descriptive study [7]. The parameters used were an estimated proportion of 0.5, a confidence level of 99%, and a desired precision of 0.05 for a finite population of 199.

Ethical consideration

Ethical approval for the study was obtained from the Shifa International Hospital Institution’s Review Board (IRB No: 0277-23). Participants provided informed consent, and their involvement was entirely voluntary. All data were kept confidential, stored securely in password-protected files, and accessed only by the research team.

Inclusion and exclusion criteria

A convenience sampling technique was applied for data collection, and participants were assured of the confidentiality of their data. Faculty members who had participated in at least one FDP were included, while those without prior FDP exposure were excluded.

Data collection

A structured and validated questionnaire (validated by five experts in the field) was used to gather data. The panel of experts included senior faculty members with expertise in medical education and research methodology. They evaluated the questionnaire for relevance, clarity, and comprehensiveness of each item. Based on their feedback, minor modifications were made to improve wording and alignment with study objectives. A pilot test was then conducted with 10 faculty members (not included in the final sample) to assess clarity and response consistency. Reliability analysis demonstrated high internal consistency with a Cronbach’s alpha of 0.822​. The content validity index (CVI) was determined for each item individually as the I-CVI and for the entire scale as the S-CVI. The I-CVI values ranged from 0.83 to 1, while the S-CVI for the questionnaire was found to be 0.94. The first section of the questionnaire (Appendix 1) covered demographic details, and faculty perceptions of FDPs were recorded in section 2 of the questionnaire across five domains: institutional benefit, career development, competency motives, professional development, and faculty resilience and innovation. Each item was rated on a 4-point Likert scale (1 = Disagree, 2 = Unsure, 3 = Partially Agree, 4 = Agree). This 4-point scale was selected to reduce neutral responses and encourage more definitive opinions. The option “Unsure” was included to capture uncertainty or lack of sufficient experience to respond, while “Partially Agree” was designed to reflect moderate agreement without complete endorsement of the statement.

Data analysis

Data were entered and analyzed by SPSS Version 22.0 (IBM Corp., Armonk, NY). Descriptive statistics, including frequencies, percentages, means, and medians, were calculated to summarize demographic data and faculty perceptions. The data were not normally distributed, as assessed by the Shapiro-Wilk test of normality (p < 0.05). The scores for individual questions across the five domains were aggregated to obtain total scores, allowing for comparisons. Faculty members were categorized based on experience, level of education, and gender. Competency scores were used to assess perceptions of teaching performance. It was assessed on five questions, with a total possible score ranging from 5 to 20 (across five items). A cutoff score of >15 was selected to reflect a clear trend toward full agreement across most items. Respondents with scores greater than 15 were categorized as "Yes, improved teaching performance,” indicating a tendency toward full agreement. Those with scores of 15 or less were grouped as "No," reflecting partial agreement or lower. Dichotomization helps simplify the interpretation of results and is particularly useful for identifying patterns in faculty who perceive a significant improvement in teaching performance versus those who do not. The faculty members were divided into two groups based on the years of experience with a cutoff value of seven years based on the eligibility criteria of the National Faculty Development Program (NFDP) in Pakistan coupled with the median years of experience in our sample approximating seven years, providing a robust basis for distinguishing between early-career and more experienced faculty member [8]. The perception of teaching performance was then analyzed using the chi-square test and binary logistic regression. A p-value <0.05 was considered significant.

## Results

Demographic information is provided in Table 1. A total of 153 faculty members participated in the study, with a predominance of female respondents (59.5%). The majority were aged between 31 and 50 years, held postgraduate degrees (87.6%), and were employed in private organizations (75.8%). Most participants (63.4%) had more than seven years of teaching experience.

**Table 1:** Demographic characteristics of respondents (n = 153) n = number of participants, data presented as percentage and frequency.

Description	Frequency	Percentage
Age		
20-30 years	8	5.20
31-40 years	66	43.10
41-50 years	53	34.60
51-60 years	17	11.10
Above 60 years	9	5.90
Gender		
Male	62	40.50
Female	91	59.50
Level of Education		
Graduates	21	13.70
Postgraduates	132	86.30
Professional Experience		
Up to 7 years	56	36.60
>7 years	97	63.40

As detailed in the methodology, non-parametric tests (Mann-Whitney U) were used due to non-normal data distribution to analyze faculty self-perception scores across the five key domains (Questionnaire Appendix 1), Institutional Benefit Orientation, Career Development, Professional Development, Competency Motives, and Faculty Innovation and Resilience, according to gender, professional experience, and level of education. Significantly higher scores were observed among men compared to women in the domains of career development, professional development, and competency motives (Table 2). 

**Table 2:** Comparison of self-reported faculty scores by Mann-Whitney U-test stratified by gender (n = 153) n = number of participants, and the p-value is calculated using the Mann-Whitney U-test. *Significant p-value, p-value <0.05 is considered significant.

Variable	Mean Ranks		p-Value	U-value
	Male (n = 62)	Female (n = 91)		
Institutional Benefit Orientation	78.89	75.71	>0.05	2704.00
Career Development	87.17	70.07	<0.05*	2190.50
Professional Development	90.87	67.55	<0.01*	1961.00
Competency Motives	89.31	68.62	<0.01*	2058.00
Faculty Innovation & Resilience	80.15	74.85	>0.05	2625.50

Grouping for professional experience was based on predefined criteria aligned with national standards and sample characteristics (as described in the Data Analysis section). When stratified by professional experience, faculty with more than seven years of teaching reported significantly higher scores in the domains of career development, professional development, and competency-related perceptions (p < 0.001), as shown in Table 3. 

**Table 3:** Comparison of self-reported faculty scores by the Mann-Whitney U-test stratified by professional experience (n = 153) n = number of participants, and p-value is calculated by the Mann-Whitney U-test. *Significant p-value, p-value <0.05 is considered significant.

Variable	Mean Ranks		p-Value	U-value
	Up to 7 years (n = 56)	> 7 years (n = 97)		
Institutional Benefit Orientation	76.58	77.25	>0.05	2692.00
Career Development	28.50	105.00	<0.001*	0.00
Professional Development	28.50	105.00	<0.001*	0.00
Competency Motives	30.76	103.70	<0.001*	126.50
Faculty Innovation & Resilience	75.27	78.00	>0.05	2619.00

Comparison of scores across groups with different educational levels revealed no statistically significant differences in most domains, except for faculty innovation and resilience, where more experienced faculty scored higher (Table 4).

**Table 4:** Comparison of self-reported faculty scores by the Mann-Whitney U-test stratified by level of education (n = 153) n = number of participants, and the p-value is calculated by the Mann-Whitney U-test. *Significant p-value, p-value <0.05 is considered significant.

Variable	Mean Ranks		p-Value	U-value
	Graduates (n = 21)	Postgraduates (n = 132)		
Institutional Benefit Orientation	71.16	77.83	>0.05	1162.00
Career Development	64.16	78.82	>0.05	1029.00
Professional Development	64.89	78.72	>0.05	1043.00
Competency Motives	65.18	78.68	>0.05	1048.50
Faculty Innovation & Resilience	64.97	78.71	<0.05*	1044.00

Chi-square analysis demonstrated a strong and statistically significant association between perception of teaching performance and professional experience (χ² = 116.823, p < .001). Gender was also significantly associated with performance (p < 0.01), while level of education showed no significant association (Table 5).

**Table 5:** Chi-square association of teaching performance with selected variables p-Value is calculated by the chi-square test. *Significant p-value, p-value <0.05 is considered significant.

Variable	Chi-square	df	p-Value
Professional Experience	116.823	1	<0.001*
Gender	7.608	1	<0.01*
Level of Education	2.382	1	>0.05

Binary logistic regression analysis (Table 6) showed that professional experience was a significant predictor of teaching performance after adjusting for gender and level of education (OR = 0.004, 95% CI = [0.001-0.016], p < 0.001) with more experienced faculty demonstrating markedly higher odds of perceiving FDPs as beneficial to teaching performance (Exp(B) = 0.004).

**Table 6:** Binary logistic regression predicting teaching performance p-Value is calculated by binary logistic regression. *Significant p-value, p-value <0.05 is considered significant.

Variable	B	SE	Wald	p-Value	Exp(B) (Odds Ratio)	95% CI
Professional Experience	-5.970	0.693	62.489	<0.001*	0.003	0.001-0.016

## Discussion

This study assessed participants’ self-perceptions of the effectiveness of FDPs across five key domains and compared these perceptions based on demographic variables like gender, educational level, and teaching experience. Most respondents demonstrated favorable perceptions toward FDPs, particularly in the domains of institutional benefit, competency motives, and faculty resilience. The findings align with previous research highlighting the role of well-structured FDPs in contributing positively to teaching practices, faculty confidence, and overall institutional success [9,10]. Notably, faculty members with more than seven years of teaching experience reported significantly higher scores in the domains of career development, professional development, and competency motives. Their perceptions differed significantly regarding the impact of FDPs on teaching performance, a finding consistent with existing literature, which suggests that experienced educators often derive greater benefit from advanced pedagogical training due to their broader contextual understanding and practical exposure [11]. As faculty advance in their careers, they may increasingly recognize the strategic value of FDPs for both academic advancement and improvement in teaching effectiveness. These findings are supported by studies indicating that experienced educators often exhibit higher readiness for adopting educational innovations and are more reflective in their practice [12].

Interestingly, in the current study, gender differences were observed in the domains of career and professional development, with male faculty reporting higher median scores. This may be due to gender-specific challenges encountered in accessing professional development opportunities and balancing work-life demands. This may be attributed to gender-specific challenges in accessing professional development opportunities and balancing work-life responsibilities. These findings align with previous studies, which highlight that female faculty often encounter systemic and structural barriers that can hinder their participation in career-advancing activities [13-15]. Therefore, when planning FDPs, it is important to incorporate gender-sensitive elements to ensure equitable access and benefits for all faculty members and to enhance inclusivity. Future FDPs should consider implementing flexible formats (e.g., hybrid sessions), incorporating mentorship models tailored to female faculty needs, and fostering a supportive institutional culture that actively addresses gender-related barriers. In contrast, the level of education did not significantly influence perceptions of the impact of FDPs on teaching performance, as the scores showed no significant differences, findings that are consistent with previous research [16]. This suggests that formal academic qualifications alone may not determine the effectiveness of FDP participation; instead, experiential learning and ongoing professional engagement may play a more substantial role. It also supports the growing view that continuing education through FDPs provides dynamic, practice-oriented skills that complement traditional academic training [10].

The scores for the domain of faculty resilience and innovation were consistently high across all demographic groups, indicating a shared recognition of FDPs as catalysts for adaptability and innovation. This finding is particularly relevant in today’s evolving educational landscape, where faculty members face rapid technological and pedagogical changes. Participants in the study agreed that FDPs enhance their ability to meet these challenges effectively, improve adaptability, and serve as a cornerstone for fostering a culture of continuous improvement. The findings align with Sorcinelli’s 50-year retrospective study, which highlights FDPs as pivotal in building faculty self-efficacy and promoting innovation [17]. Another study has also reported the role of FDPs as instrumental in helping faculty navigate educational change, adopt new teaching strategies, and build capacity for adaptability [11]. It was interesting to note that the participants of the current study overwhelmingly perceived FDPs as contributing positively to institutional growth and academic excellence. Participants highlighted that FDPs enhance the ability to share best practices and promote the institution’s academic reputation. There was a consensus that FDPs help position the institution competitively at the national and international levels. Institutions actively support FDPs to enhance their academic image and reputation. These findings are consistent with previous research, which indicates that well-structured FDPs not only enhance individual faculty competence but also contribute to elevating an institution’s academic reputation and overall competitiveness at both national and international levels [18]. The results are reinforced by a study by Swanwick T, who emphasized that institutional support for FDPs strengthens faculty engagement and institutional outcomes [19].

While the findings of the present study underscore the value of FDPs, there are certain limitations that should be acknowledged more explicitly. The cross-sectional nature of the study restricts causal interpretations, and the convenience sampling approach, although suitable for the descriptive nature of the study and allowing timely access to the target population, may introduce selection bias and limit the broader application of the findings. The reliance on self-reported data may also lead to response bias. Additionally, as a single-center study, its generalizability may be limited in academic settings with different organizational cultures or levels of resource availability. Future research should explore longitudinal study designs to assess the sustained impact of FDPs over time, including actual improvements in teaching practices and student learning outcomes. Multi-institutional studies across varied geographic and institutional contexts are also needed to validate and extend the current findings. Moreover, qualitative studies exploring faculty narratives can offer deeper insights into how gender, experience, and institutional support interact to shape perceptions and experiences of FDPs.

## Conclusions

In conclusion, this study contributes to the growing body of evidence supporting the effectiveness of FDPs in medical education. FDPs appear to play a significant role in enhancing faculty competence, supporting career and professional growth, fostering innovation, and ultimately improving teaching performance, especially among experienced educators. Male faculty members have a strong perception of the effectiveness of these programs as compared to females. Moreover, Faculty members perceive FDPs as essential for institutional reputation and excellence. Institutes implement these programs not just to improve teachers’ competence but to compete at both national and international levels. The findings highlight the need for sustained institutional investment in context-sensitive, inclusive, and outcome-driven faculty development strategies to meet the evolving demands of medical education. Future research should consider longitudinal, multi-center designs to assess the sustained impact of FDPs on teaching outcomes and institutional growth. Qualitative studies could also explore faculty perceptions in greater depth, particularly those of underrepresented groups.

From a practical standpoint, institutions, particularly in resource-limited settings, should prioritize accessible and contextually relevant FDPs. Recommendations include offering hybrid delivery formats, integrating mentorship components, and incorporating gender-sensitive policies to ensure equitable benefits. Institutional policies should focus on continuous support and structured feedback mechanisms to promote long-term faculty engagement and teaching effectiveness.

## Appendices

**Table 7:** Questionnaire: Self-perception of faculty development program in medical institution

Section 1: Demographic information
Age:
Gender:
Marital Status:
Level of Education:
Professional Experience:
Section 2: Perceptions across five Domains:
Institution Benefit Orientation
I believe FDPS are essential to meet the evolving needs of the healthcare and medical education sector.
FDPs enhance the ability to share best practices and promote institution’s academic reputation.
FDPs contribute directly to the institution’s growth and academic excellence.
I believe FDPs help position the institution competitively at the national and international levels.
Management actively supports faculty development programs to enhance the institution’s academic image and reputation.
Career Development
FDPs contribute to my personal growth and enhance my subject knowledge.
FDPs improve my professional skills and advance my academic career.
Participation in FDPs helps me fulfill institutional and regulatory career progression requirements.
Innovation & Faculty Resilience
FDPs improve my ability to adapt to changing educational and institutional demands.
FDPs encourage the adoption of innovative teaching methods and technologies.
Competency Motives
My teaching and overall academic performance have improved due to FDPs
I feel that my teaching skills and pedagogical competencies have been enhanced through FDPs
I am able to effectively regulate my emotions in classroom and academic settings.
I feel confident in fulfilling both my academic and administrative responsibilities.
FDPs are essential for enhancing teaching performance.
Professional Development
FDPs guide me in the right direction for my professional growth.
FDPs support the achievement of my long-term professional goals.
FDPs encourage continuous learning and self-improvement.

## References

[1] Chalmers D, Gardiner D (2015). The measurement and impact of university teacher development programs. Educar.

[2] Gade S, Mishra V (2020). Impact analysis of the National Faculty Development Program for Medical Teachers: A way forward. J Krishna Inst Med Sci Univ.

[3] Musset P (2010). Initial teacher education and continuing training policies in a comparative perspective: Current practices in OECD countries and a literature review on potential effects. OECD Education Working Papers, No. 48.

[4] Chahar B, Jain SR, Hatwal V (2021). Mediating role of employee motivation for training, commitment, retention, and performance in higher education institutions. Probl Perspect Manag.

[5] Guglielmo BJ, Edwards DJ, Franks AS (2011). A critical appraisal of and recommendations for faculty development. Am J Pharm Educ.

[6] Gauthier L, Waqar Y (2021). High impact learning for facilitator training and development. Int J Scholar Teach Learn.

[7] (2025). OpenEpi: Open source epidemiologic statistics for public health. https://www.openepi.com/Menu/OE_Menu.htm.

[8] (2025). Higher Education Commission. (n.d.). National Faculty Development Program (NFDP). National Academy of Higher Education (NAHE), Pakistan. https://www.hec.gov.pk/english/nahe/pages/nfdp.aspx.

[9] Raza SA, Naqvi AH, Lodhi AS (2011). Assessing need for teaching development of faculty at universities of Pakistan: A students’ perspective. Bull Educ Res.

[10] Bilal Bilal, Guraya SY, Chen S (2019). The impact and effectiveness of faculty development program in fostering the faculty's knowledge, skills, and professional competence: A systematic review and meta-analysis. Saudi J Biol Sci.

[11] Efu SI (2022). Reflection: A means to faculty engagement in meaningful continuing professional development. Teach Dev.

[12] Steinert Y, Naismith L, Mann K (2012). Faculty development initiatives designed to promote leadership in medical education. A BEME systematic review: BEME Guide No. 19. Med Teach.

[13] Cutter CM, Griffith KA, Settles IH, Stewart AJ, Kerr EA, Feldman EL, Jagsi R (2024). Gender differences in faculty perceptions of mentorship and sponsorship. JAMA Netw Open.

[14] Bickel J, Wara D, Atkinson BF (2002). Increasing women's leadership in academic medicine: Report of the AAMC Project Implementation Committee. Acad Med.

[15] Eiland LS, Shields KM, Smith SE, Covington EW, Edwards A, Kinney SR, Haines SL (2021). Development and assessment of a nationwide, cross-discipline women faculty mentoring program. Curr Pharm Teach Learn.

[16] Karahoca D, Kurnaz A ( 2014). Qualification perception of academics in universities for innovation management. Procedia Soc Behav Sci.

[17] Sorcinelli MD ( 2020). The evaluation of faculty development programs in the United States: A fifty-year retrospective (1970s-2020). Excell Innov Teach Learn.

[18] Alhassan AI (2022). Implementing faculty development programs in medical education utilizing Kirkpatrick's model. Adv Med Educ Pract.

[19] Swanwick T (2008). See one, do one, then what? Faculty development in postgraduate medical education. Postgrad Med J.

